# The efficacy of botulinum toxin type A treatment and surgery for acute acquired comitant esotropia

**DOI:** 10.3389/fmed.2023.1219419

**Published:** 2023-08-04

**Authors:** Yipao Li, Luyao Tong, Yuanyuan Chen, BinJun Zhang, Minghui Wan, Xiangping Yin, Fang Zhang

**Affiliations:** ^1^National Clinical Research Center for Ocular Diseases, Eye Hospital, Wenzhou Medical University, Wenzhou, China; ^2^The First Affiliated Hospital of Ningbo University, Ningbo, China

**Keywords:** acute acquired comitant esotropia, AACE, botulinum toxin type A, BTXA, surgical outcome acute acquired comitant esotropia, surgical outcome

## Abstract

**Aim:**

To compare the long-term efficiency of botulinum toxin type A (BTXA) injection and surgery on acute acquired comitant esotropia (AACE).

**Methods:**

This retrospective study enrolled patients with AACE from January 2020 to August 2022. The horizontal angle of deviation pre- and post-treatment was measured. Deviations in BTXA and surgical treatment were compared. The BTXA group was divided into adequate treatment (AT) and inadequate treatment (inAT) subgroup based on the deviation of no more than 4 prism diopters (at near and distance) or temporary exotropia at the 2 week follow-up. The two subgroups were compared to determine the long-term efficacy of BTXA treatment.

**Results:**

Ninety-two patients with AACE were included. Follow-up was 6 months. The deviations of the surgery and BTXA group were significantly smaller at the 6 month follow-up than at pre-treatment (*p* < 0.001). The deviation before treatment in the surgery group was larger than in the BTXA groups (*p* < 0.001) but smaller at the 6 month follow-up (*p* < 0.001). The deviation was similar in the AT-BTXA and inAT-BTXA subgroups before treatment (*p* = 0.322 for distance and *p* = 0.051 for near) but smaller in the AT-BTXA subgroup at 6 month follow-up (*p* < 0.001 for near and distance).

**Conclusion:**

Surgery and BTXA successfully treat AACE. Surgery has a more precise and lasting therapeutic effect than BTXA. AACE patients adequately treated with BTXA and with deviations of no more than 4 prism diopters at 2 weeks follow-up had better outcomes.

## 1. Introduction

Acute acquired concomitant esotropia (AACE) is a comitant esotropia characterized by acute onset diplopia in older children and adults (1–3). Excessive near-work and anatomical abnormalities may lead to excessive convergence and induce AACE (4–7). The effects of strabismus surgery and botulinum toxin type A (BTXA) on AACE have been investigated in several studies. Both treatments effectively reduce the degree of strabismus and restore stereoscopic vision (5, 8–10). Accurate surgery dose can be designed according to the pre-surgery deviation (11). BTXA reduces deviation by temporarily blocking the neuromuscular junction without surgical complications (12). Though the efficiency of BTXA and surgery on AACE has been explored, there is no consensus on whether BTXA can achieve the same success rate as surgery (13–15).

The BTXA treatment is thought to cause less injury and incur fewer costs than other treatments; nevertheless, it is associated with a higher recurrence rate at follow-up (14, 16, 17). BTXA metabolism lasts months, making it challenging to speculate on the long-term (6 months) efficacy in the early follow-up (12). The accurate BTXA dose is also challenging to calculate based on the deviation because of the individual response of BTXA (18). These factors may delay subsequent therapy of recurrence, as it requires a 6 month follow-up to confirm efficacy. An index in the early follow-up period to speculate on the efficacy at 6 months post-injection needs to be developed.

Therefore, we conducted this study to compare the efficacy of BTXA and surgery and to examine the early indexes of long-term BTXA efficacy.

## 2. Methods

The Ethics Committee of Wenzhou Medical University approved this retrospective study (Approval ID. 2020-148-K-133-01), which adhered to the tenets of the Declaration of Helsinki. Written informed consent was waived because the study was retrospective with anonymized data.

We included patients with AACE from January 2020 to August 2022 at the Affiliated Eye Hospital of Wenzhou Medical University. The inclusion criteria were (1) sudden-onset diplopia, diagnosed with AACE; (2) history of BTXA injection or surgery. The exclusion criteria were (1) history of eye disease, ocular surgery, or ocular trauma; (2) intracranial or neurologic disease; (3) follow-up of less than 6 months. All enrolled patients were examined, treated, and followed by the same ophthalmologist.

The horizontal angle of deviation pre-and post-treatment, with refractive correction, was measured with the prism and cover tests at 6 m (distance) and 40 cm (near) fixation. The spherical equivalents in each eye were averaged and recorded as the value of refractive errors. Ophthalmic examination by ophthalmologists and neurological examination by neurologists were performed to rule out ocular, intracranial, and neurologic diseases. After administration of topical anesthesia, BTXA (Hengli, China) injection (30 gauge × 1/2 in) was performed at about 6 mm posterior to the medial rectus insertion without conjunctival incision and electromyography guidance. The injection doses were 4.0 units for a deviation of more than 35 PD, 3.5 units for 26 PD to 35 PD, and 3.0 units for 10 PD to 25 PD. Unilateral medial rectus recession (no more than 20 PD) or medial rectus recession combined with lateral rectus resection (more than 20 PD) were performed under general anesthesia (patients less than 14 years old) or local anesthesia (patients at least 14 years old).

Patients were divided into a BTXA group and a surgery group. Patients with a deviation of no more than 4 PD can achieve a diplopia-free state by self-control. The patients in both groups who achieved a deviation of no more than 4 PD (at near and distance) or temporary exotropia at the 2 week follow-up were considered adequately treated. The groups were then divided into an adequate treatment (AT) group and an inadequate treatment (inAT) group. Deviation pre-and post-treatment in groups were compared. At the 6 month follow-up, the absence of diplopia throughout the day and horizontal deviation of no more than 8 PD (both at distance and near) was considered a successful treatment. Statistical analyses were performed with SPSS version 26.0 (SPSS, Inc., Chicago, IL, United States). Data were compared using the Mann–Whitney U-test. Spearman’s correlation coefficient was calculated to explore relationships between two factors. Differences where *p* < 0.05 were considered statistically significant.

## 3. Results

We included 92 patients (64 males and 28 females) with AACE, of whom 51 were treated with BTXA and 41 with surgery. All patients were Chinese, ranging from 6 to 50 years old (mean 23.9 years), and had diplopia for 1 month to 10 years (median 1 year).

The clinical characteristics of the two treatment groups are displayed in Table 1 and the deviation in the BTXA group was smaller before treatment (*p* < 0.001) but was larger at the 6 month follow-up than in the surgery group (*p* < 0.001). The success rate was lower in the BTXA group at the 6 month follow-up (*p* < 0.001). At the 2 week follow-up, there was no significant difference between the groups in deviation examined at near (*p* = 0.908) and distance (*p* = 0.452). Compared with deviation before treatment, the deviations of the surgery group (*p* < 0.001 at near and distance) and BTXA group (*p* < 0.001 at near and distance) were significantly smaller at the 6 month follow-up.

**Table 1:** Clinical characteristics of the BTXA group and surgery group.

Characteristic	BTXA Group (*n* = 51)	Surgery Group (*n* = 41)	*p*-value^*^
Spherical equivalent (diopters) Pre-treatment	−4.50 (−6.50–−3.25)	−4.00 (−5.50–−0.75)	0.113
Deviation in primary position (PD)			
Pre-treatment			
Distance	20 (15–30)	40 (25–45)	<0.001
Near	18 (12.5–25)	35 (25–40)	<0.001
2 week follow-up			
Distance	0 (−5.5–4)	0 (0–0)	0.908
Near	0 (−4.5–2)	0 (0–0)	0.452
6 month follow-up			
Distance	6 (4–14)	0 (0–0)	<0.001
Near	4 (2–8)	0 (0–0)	<0.001
Absence of diplopia at 6 month follow-up	58.8% (30/51)	95.1% (39/41)	<0.001

Data are presented as range and median (interquartile range, 25th to 75th percentile). BTXA, botulinum toxin type A; PD, prism diopter. ^*^Mann–Whitney U test. The absence of diplopia means patients worked and lived without diplopia throughout the day.

BTXA metabolism occurs over time, and its effect develops slowly. We focused on the 2 week follow-up interval, which showed no significant difference in deviation between the two groups. We identified patients in both groups who achieved a deviation of no more than 4 PD (at near and distance) or temporary exotropia at the 2 week follow-up and were considered adequately treated at that time. The clinical characteristics are displayed in Table 2. At the 2 week follow-up, the deviation was similar at distance (*p* = 0.076) in the two groups and smaller at near in the BTXA group (*p* = 0.007). However, the deviations were larger at near and distance in the BTXA group at the 6 month follow-up (*p* < 0.001). The success rate was lower in the BTXA group at the 6 month follow-up (*p* < 0.001). In the BTXA group, the deviation at near and distance at the 6 month follow-up was associated with the deviation at near (*p* = 0.001, *r* = 0.447) and distance (*p* < 0.001, *r* = 0.529) at the 2 week follow-up. In the BTXA group, the success rate at the 6 month follow-up was significantly associated with deviation at near (*p* < 0.001, *r* = −0.516) and distance (*p* < 0.001, *r* = −0.529) at the 2 week follow-up. At the 6 month follow-up, the patients in BTXA group with deviation of no more than 8 PD were free of diplopia.

**Table 2:** Clinical characteristics of the adequately treated patients in two groups.

Characteristic	BTXA Group (*n* = 41)	Surgery Group (*n* = 40)	*p*-value^*^
Spherical equivalent (diopters) Pre-treatment	−4.5 (−6.50–−3.50)	−3.5 (−5.375–−0.625)	0.070
Deviation in primary position (PD)			
Pre-treatment			
Distance	20 (15–25)	37.5 (25–45)	<0.001
Near	16 (12–20)	32.5 (25–40)	<0.001
2 week follow-up			
Distance	0 (−8–2)	0 (0–0)	0.076
Near	0 (−6–0)	0 (0–0)	0.007
6 month follow-up			
Distance	6 (3–9)	0 (0–0)	<0.001
Near	4 (2–5)	0 (0–0)	<0.001
Absence of diplopia at 6 month follow-up	73.2% (30/41)	97.5% (39/40)	<0.001

Data are presented as median (interquartile range, 25th to 75th percentile). BTXA, botulinum toxin type A; PD, prism diopter. ^*^Mann–Whitney U test. “Adequate” means patient without diplopia. Adequate treatment means patients achieved a deviation of no more than 4 PD (at near and distance) or temporary exotropia at the 2 week follow-up.

We divided the BTXA group into the inAT-BTXA and AT-BTXA subgroups based on the deviation at the 2 week follow-up. The two groups’ deviations at distance (*p* = 0.322) and near (*p* = 0.051) before treatment were similar. At the 6 month follow-up, deviations were significantly larger in the inAT-BTXA subgroup at near (*p* < 0.001) and distance (*p* < 0.001), as displayed in Table 3. Among the AT-BTXA subgroup, the success rate was 100% in patients with no more than 15 PD deviations, 83.3% with no more than 25 PD, and 72.3% with no more than 35 PD.

**Table 3:** Clinical characteristics of the AT-BTXA and inAT-BTXA subgroups.

Characteristic	AT-BTXA Group (*n* = 41)	inAT-BTXA Group (*n* = 10)	*p*-value^*^
Spherical equivalent (diopters)			
Pre-treatment	−4.50 (−6.50–−3.50)	−4.00 (−4.00–−3.00)	0.263
Deviation in primary position (PD)			
Pre-treatment			
Distance	20 (15–25)	27.5 (16–35)	0.322
Near	16 (12–20)	25 (16–30)	0.051
2 week follow-up			
Distance	0 (−8–2)	10 (8–14)	<0.001
Near	0 (−6–0)	9 (4–10)	<0.001
6 month follow-up			
Distance	6 (3–9)	18 (14–25)	<0.001
Near	4 (2–5)	14 (10–20)	<0.001
Absence of diplopia at 6 month follow-up	73.2% (30/41)	0.0% (0/10)	<0.001

Data are presented as median (interquartile range, 25th to 75th percentile). AT, adequate treatment; inAT, inadequate treatment; BTXA, botulinum toxin type A; PD, prism diopter. ^*^Mann–Whitney U test.

Complications of the BTXA-injected eye, including temporary exotropia and ptosis, were relieved or resolved during the follow-up. No serious severe complications were found in the surgery group, except for intraoperative bleeding.

## 4. Discussion

This study compared the treatment efficacy of BTXA and surgery at long-term follow-up and revealed that surgery has a more precise and lasting therapeutic effect with a success rate of 95.1%. We also observed that adequate BTXA treatment, with no more than 4 PD deviations at 2 weeks follow-up, was associated with better outcomes.

As the primary treatment of strabismus, surgery was found to be safe and effective in improving ocular alignment, eliminating diplopia, developing binocular fusion, and expanding binocular visual fields (19). Careful surgical planning and operation can prevent severe complications, including scleral perforations, orbital inflammation, muscle slip, and anesthesia complications (20). BTXA was also found to be a safe, effective, and repeatable treatment for AACE, with fewer iatrogenic injuries (10, 17, 21, 22). Because of the individual response of BTXA (18), and limitations of the syringe scale, surgeons cannot precisely administer BTXA doses according to the deviation. Therefore, each BTXA dose corresponds to a range of deviations, which differ from surgery. By comparing the efficiency of the two treatments on AACE, surgery has a similar or better success rate (13–15). In the present study, surgery and BTXA significantly improved ocular alignment at the final follow-up. We then compared the clinical characteristics of the treatment groups. The deviation was more significant in the surgery group before treatment but significantly smaller at the 6 month follow-up, and the success rate was also more significant at the 6 month follow-up. Considering the time required for the BTXA effect and the metabolism, we identified patients in both groups who were adequately treated at the 2 week follow-up. The deviation was also more significant in the BTXA group at the 6 month follow-up. These findings suggest that surgery is more effective, precise, and durable than BTXA.

The BTXA injection is an alternative treatment for AACE. The success at 6 month follow-up ranged from 45 to 90.6% (10, 13–15, 21, 22). The substantial variability in success rates suggests the instability of BTXA in the treatment of AACE. Several months of BTXA metabolism causes a gradual decrease in success rate, which results in a delayed determination of final treatment success after injection (12, 14, 16, 17). It would be beneficial to identify an indicator in the early stage that determines whether the treatment is successful. In this study, the deviation and success rate of the BTXA group at the 6 month follow-up was associated with the deviation at the 2 week follow-up. Based on the deviations at the 2 week follow-up, the BTXA group was divided into AT-BTXA and the-BTXA subgroups. The deviations before treatment were similar in the two groups. At the 6 month follow-up, deviations were significantly larger in the inAT-BTXA subgroup. These results suggest that patients with a deviation of no more than 4 PD after 2 weeks of BTXA injection have a significantly better outcome. A deviation of more than 4 PD at the 2 week follow-up can indicate unsuccessful treatment 6 months after BTXA injection.

Quantitative evidence suggested that augmented-surgery doses should be performed in AACE to obtain a satisfactory outcome (23). In this study, all patients underwent surgery before this concept was proposed; 95.1% of patients were satisfied, and there was no diplopia.

The retrospective and non-randomized design are the primary limitations of this study. Randomized controlled clinical trials need to be designed to increase the evidence strength. The follow-up in this study was 6 months. Longer follow-up will provide more data on treatment efficacy. The surgery group achieved better outcomes with greater deviation before treatment. Though the deviation was different before treatment in both groups, this still indicated a better outcome of surgery.

In conclusion, both surgery and BTXA are efficient for AACE. Surgery has a more precise and lasting therapeutic than BTXA. AACE patients who were adequately treated with BTXA with deviations of no more than 4 PD at 2 weeks follow-up had better outcomes.

## Data availability statement

The raw data supporting the conclusions of this article will be made available by the authors, without undue reservation.

## Ethics statement

The studies involving human participants were reviewed and approved by Ethics Committee of Wenzhou Medical University. Written informed consent from the participants’ legal guardian/next of kin was not required to participate in this study in accordance with the national legislation and the institutional requirements.

## Author contributions

YL and FZ conceived and designed the study. YL performed the treatments and follow up with the patients. YL and LT conducted the acquisition and statistical analysis of the data. YL, LT, YC, MW, BZ, and XY drafted the manuscript. FZ supervised the study and critically revised the manuscript. All authors contributed to the article and approved the submitted version.

## Conflict of interest

The authors declare that the research was conducted in the absence of any commercial or financial relationships that could be construed as a potential conflict of interest.

## Publisher’s note

All claims expressed in this article are solely those of the authors and do not necessarily represent those of their affiliated organizations, or those of the publisher, the editors and the reviewers. Any product that may be evaluated in this article, or claim that may be made by its manufacturer, is not guaranteed or endorsed by the publisher.
